# Therapeutic Effects of Stromal Stem Cells Derived from Ovarian Tissue in a Cyclophosphamide-Induced Rat Model of Ovarian Failure

**DOI:** 10.1007/s43032-026-02114-7

**Published:** 2026-04-29

**Authors:** Mehpare Akgün, Murat Serkant Ünal, Faruk Altınbaşak, Elif Önder, Semih Tan

**Affiliations:** 1 Denizli State Hospital, Denizli, Turkey; 2 Department of Histology and Embryology, Faculty of Medicine, Pamukkale University, Denizli, Turkey; 3 Department of Histology and Embryology, Faculty of Medicine, Hitit University, Çorum, Turkey; 4 Department of Histology and Embryology, Faculty of Medicine, Ordu University, Ordu, Turkey

**Keywords:** Cell culture techniques, Stromal cells, Premature ovarian failure, Cyclophosphamide, Stromal stem cell

## Abstract

Chemotherapeutic agents used in cancer treatment cause damage to both oocytes and granulosa cells, resulting in follicle loss and consequently premature ovarian failure. In this study the animals were randomly assigned to three groups: control (*n* = 6), chemotherapy (*n* = 6), and stem cell treatment (*n* = 6). Ovarian failure was induced in the chemotherapy and stem cell groups by intraperitoneal administration of cyclophosphamide (200 mg/kg) on days 1 and 8. Ovarian stromal stem cells (OSSCs) were isolated from the ovaries of 4-week-old donor rats (*n* = 2) using the explant culture method. On day 9, isolated stromal cells were transplanted bilaterally into the ovaries of rats in the stem cell group. Surface markers were analyzed by flow cytometry in OSSCs, and their adipogenic, osteogenic, and chondrogenic differentiation potentials were evaluated under appropriate in vitro conditions. The number of follicles and the morphological features of the ovarian tissue were histologically examined using hematoxylin and eosin (H&E) staining. Caspase 3 expression in ovarian tissue was analyzed using TUNEL and immunohistochemistry methods. Flow cytometry analysis of isolated ovarian stromal cells showed that CD54, CD90 and CD45 surface markers were expressed, while CD29 was expressed at a lower level. These findings confirmed the ability of OSSCs to differentiate into adipogenic, osteogenic, and chondrogenic lineages in vitro. Follicle counting, TUNEL and immunohistochemical analysis showed that treatment with ovarian stromal stem cells significantly reduced the number of atretic follicles and increased the number of normally developing follicles in the ovaries of chemotherapy-treated rats. Our research demonstrates the therapeutic effects of (allogeneic) ovarian stromal stem cells present in their niche on ovarian toxicity induced by chemotherapy, suggesting an alternative treatment option for approaches aimed at preserving fertility.

## Introduction

Premature Ovarian Failure (POF) is a condition that typically occurs in women of reproductive age before the age of 40, characterized by elevated gonadotropin levels (FSH, LH) and low estrogen levels. Clinically, it manifests with symptoms of amenorrhea and hypoestrogenism. Various factors, including genetic, autoimmune, metabolic, infectious, and iatrogenic factors, contribute to the etiology of POF [1]. The early loss of ovarian function not only leads to severe health complications but also has significant psychosocial impacts [2].

The loss of ovarian function is primarily associated with the depletion of the primordial follicle pool or disruptions in the folliculogenesis process. The number of primordial follicles is a key indicator of ovarian reserve. While these follicles generally remain dormant, a subset is activated at the onset of puberty and enters the developmental process during each menstrual cycle. During this process, follicles sequentially mature into primary, secondary, and Graafian follicles before ovulation [3]. Although ovarian reserve naturally declines with aging, various environmental factors and diseases may accelerate this process, leading to early ovarian failure.

One of the most significant iatrogenic factors contributing to POF is chemotherapy. Chemotherapeutic agents, particularly alkylating agents such as cyclophosphamide, target rapidly dividing malignant cells but also cause substantial damage to healthy ovarian tissue. These agents have been shown to induce DNA damage in oocytes, disrupt mitochondrial function, and trigger oxidative stress mechanisms [4, 5]. Chemotherapy-induced ovarian failure disrupts the ovarian microenvironment by mechanisms such as follicular depletion and stromal fibrosis, thereby negatively affecting folliculogenesis [6]. Currently, hormone replacement therapy (HRT) is widely used in the management of POF. Although HRT helps alleviate symptoms, it is insufficient for restoring the follicular reserve [7]. Consequently, regenerative medicine approaches, particularly stem cell-based therapies, have emerged as promising alternatives for POF treatment.

Mesenchymal stem cells (MSCs) have extensive applications in regenerative medicine and have also been investigated for their potential therapeutic effects in POF models. MSCs can be isolated from various sources, including bone marrow, adipose tissue, cartilage, amniotic fluid, placenta, and endometrium, and contribute to tissue repair through their anti-inflammatory, antifibrotic, angiogenic, and immunomodulatory properties [8, 9]. Animal models have demonstrated that MSCs support folliculogenesis, increase estrogen levels, and reduce apoptotic processes [10]. However, the regenerative potential of ovarian stromal stem cells (OSSCs), which are naturally present within the ovarian microenvironment, has not yet been fully elucidated. Ovarian stromal stem cells are a population of endogenous stem cells located within the ovarian stroma, specific to this organ. Although they may exhibit characteristics similar to those of mesenchymal stem cells, they are not classified as mesenchymal stem cells. Despite sharing immunophenotypic features, they possess functions that are specific to the local signaling environment and the organ-specific microenvironment.

The focus of this study is on ovarian stromal stem cells (OSSCs), which are naturally found within the ovarian microenvironment. These cells may contribute to the restoration of ovarian function by secreting growth factors and cytokines that support follicular development. Additionally, the intrinsic presence of OSSCs within ovarian tissue may enhance their compatibility with the tissue, thereby minimizing immune responses. However, the therapeutic potential of OSSCs remains unclear in the literature, and their efficacy in chemotherapy-induced ovarian damage is still under investigation.

To date, numerous studies have explored the use of MSCs derived from bone marrow and adipose tissue in the treatment of POF. Animal models have demonstrated that MSCs promote folliculogenesis and increase estrogen levels [10]. However, the regenerative potential of ovarian stromal stem cells (OSSCs), which exist within the ovarian microenvironment, has not yet been fully established. This study aims to evaluate the therapeutic potential of OSSCs in a cyclophosphamide-induced ovarian failure model in rats. The study will investigate whether OSSCs support follicular development, suppress apoptotic processes, and restore ovarian function. The findings of this study are expected to provide important insights into the role of ovarian stromal stem cells in the treatment of premature ovarian failure.

## Materials and Methods

### Animals

Approval for the study was obtained from the Animal Experiments Ethics Committee of Pamukkale University on June 5, 2020, with the number PAUHADYEK-2020/10. In our study, a total of 18 female Wistar Albino rats, aged 8 weeks and weighing between 200 and 250 g, were used. Throughout the experimental protocol, the rats were kept in separate cages within a quiet room with a controlled temperature (21 ± 1 °C) and humidity (65–70%), under a 12-hour light-dark cycle. The animals were provided with standard rat feed and tap water ad libitum. Prior to randomization, vaginal smears were collected from all rats to determine their estrous cycle stages, and only animals in the same phase of the estrous cycle were included in the study. The rats were then randomly assigned to three groups: control group (*n* = 6), chemotherapy group (*n* = 6), and stem cell group (*n* = 6). The body weights of the animals were recorded before and after the experimental treatments. Additionally, two prepubertal (4-week-old) female rats were included in the study to obtain ovarian stromal stem cells.

### Procedure for Inducing Ovarian Toxicity with Cyclophosphamide in Rats

**Fig. 1:**
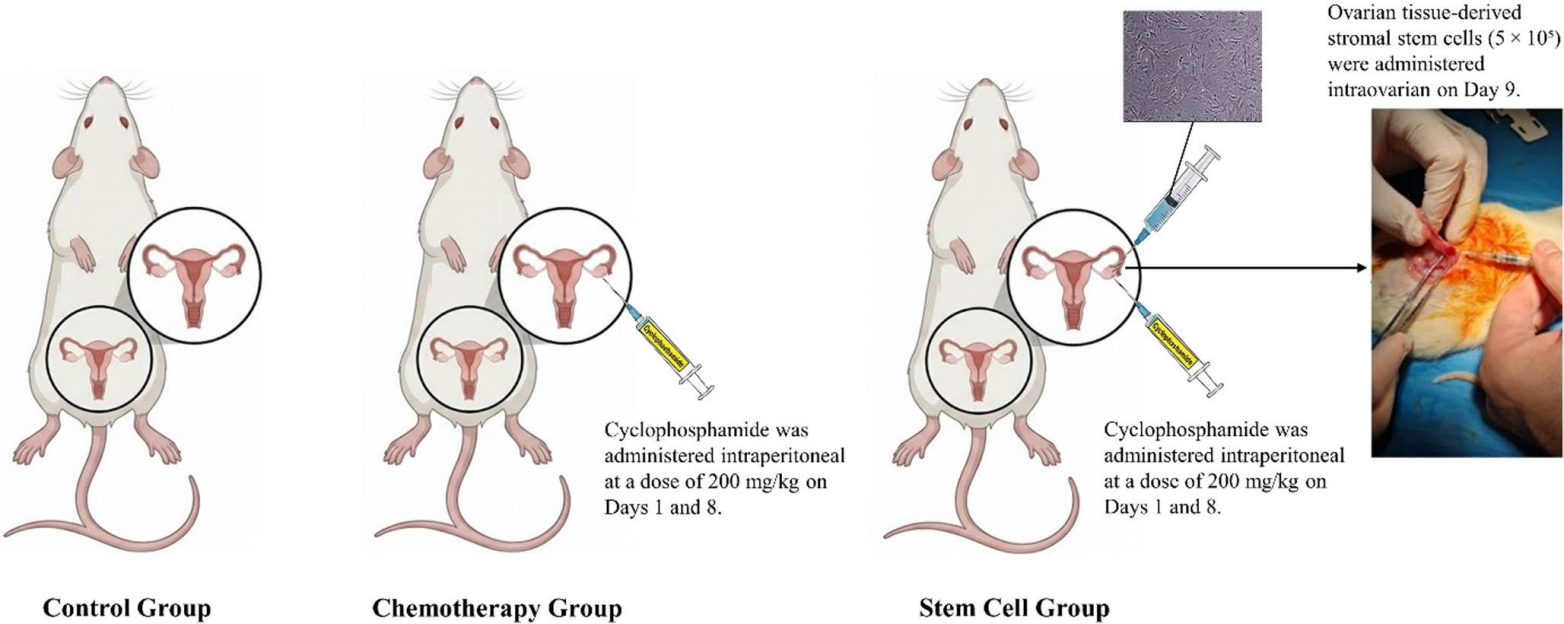
Schematic representation of cyclophosphamide-induced ovarian failure and its treatment using ovarian tissue-derived stromal stem cells in a rat model

Ovarian failure was induced by intraperitoneal administration of cyclophosphamide (CTX, Endoxan 1 g Baxter) to induce ovarian failure. The chemotherapy and the stem cell group received 200 mg/kg of cyclophosphamide dissolved in phosphate-buffered saline (PBS) on days 1 and 8 (Fig. 1) [11].

### Stem Cell Group

Ovarian-derived stromal stem cells were injected directly into both ovaries of rats in the stem cell group (*n* = 6) under general anesthesia. The injections were performed on day 9, one day after the second cyclophosphamide administration. Each ovary received 500,000 (5 × 10⁵) passage 3 OSSCs suspended in 0.01 mL of PBS (Fig. 1).

### Derivation of Stromal Stem Cells from Ovarium

Ovarian tissues from two 4-week-old female Wistar albino rats were excised under sterile conditions. The excised tissues were thoroughly cleaned of clot residues by washing them in PBS in a sterile container. The tissues were then transferred to the cell culture laboratory in a sterile solution (DMEM containing penicillin/streptomycin) and washed several more times with PBS in a laminar flow cabinet to remove any remaining blood. Subsequently, the adipose tissue surrounding the ovarian tissue was removed, and the tissue was cut into small pieces and placed in 100-mm Petri dishes [11]. The cells were incubated at 37 °C in an incubator containing 5% CO_2_. After 24 h, contamination and cell viability checks were performed. The complete culture medium was prepared by mixing 450 ml of Dulbecco’s Modified Eagle Medium (DMEM) (Capricorn Scientific, Germany), 50 ml of Fetal Bovine Serum (FBS) (Capricorn Scientific, Germany), and 5 ml of Penicillin-Streptomycin (Pan Biotech, Germany). The cells attached to the culture dishes were detached using 0.25% trypsin (Hyclone, USA) and transferred to new culture dishes containing fresh complete medium. The medium in the culture dishes was changed every 2 days to maintain optimal culture conditions. The proliferating cells were stained with trypan blue for viability assessment, and cell counts were determined using a Neubauer improved counting chamber using a light microscope. All these stages were observed using an inverted microscope (CKX41 Olympus, Japan).

### Characterization of Ovarian Stromal Stem Cells

For flow cytometry analysis, stromal stem cells at passage 2 (P2) were analyzed for the expression of surface markers CD90, CD54, CD45, CD29, and RT1D. Flow cytometric analysis was performed at Nepenthe Research Technologies Center in Kocaeli, using a Sysmex Cube 8 model device.

### In Vitro Differentiation Assays of OSSCs

The adipogenic, osteogenic, and chondrogenic differentiation potentials of OSSCs were evaluated in vitro at passage 4 (P4). Cells were seeded in 12-well culture plates and cultured until approximately 70–80% confluence. Differentiation was induced using commercially available differentiation media according to the manufacturer’s instructions.

For adipogenic differentiation, cells were cultured in StemPro™ Adipogenesis Differentiation Kit (Gibco, Invitrogen Cell Culture, Carlsbad, CA, USA). The differentiation medium was replaced every 2–3 days and cells were maintained in adipogenic conditions for 14–15 days. At the end of the induction period, cells were washed with PBS and fixed with 10% neutra-buffered formalin for 30 min at room temperature. Lipid droplet formation was detected by staining with Oil Red O solution (Sigma–Aldrich, Chemie GmbH, Taufkirchen, Germany) for 60 min. Excess dye was removed by washing with distilled water.

For osteogenic differentiation, OSSCs were cultured in StemPro™ Osteogenesis Differentiation Kit (Gibco, Invitrogen Cell Culture, Carlsbad, CA, USA). The medium was replaced twice a week and cells were maintained for 21 days. After differentiation, cells were washed with PBS and fixed in 4% paraformaldehyde for 30 min. Calcium deposition was evaluated by staining with Alizarin Red S solution (Sigma–Aldrich, Chemie GmbH, Taufkirchen, Germany) for 2–5 min.

For chondrogenic differentiation, cells were cultured in StemPro™ Chondrogenesis Differentiation Kit (Gibco, Invitrogen Cell Culture, Carlsbad, CA, USA). The medium was refreshed twice weekly and the cells were incubated for 14 days. At the end of the differentiation period, cells were fixed with 4% paraformaldehyde for 30 min and stained with Alcian Blue solution (1% in 0.1 N HCl) (Sigma–Aldrich, Chemie GmbH, Taufkirchen, Germany) for 30 min to detect glycosaminoglycan accumulation.

After staining, cells were rinsed with distilled water and examined under an inverted phase-contrast microscope (Olympus, Tokyo, Japan), and representative images were captured.

### Histological Evaluation

Two weeks after the stromal stem cell injection, the ovaries of all rats were removed, and routine light microscopy tissue examination was performed. Sections of 5 μm thickness were taken from paraffin blocks and stained with hematoxylin-eosin (H&E). The sections were histopathologically examined under a light microscope. In the 1st, 5th, and 10th sections, the numbers of primordial, primary, secondary, and tertiary follicles was counted. The sections were examined with an Olympus BX-51 light microscope and photographed with an Olympus PP72 Digital Camera.

### dUTP Biotin Nick End Labeling (TUNEL) Staining

To identify apoptotic cells in ovarian tissue, an in situ apoptosis detection kit based on the DNA fragmentation end labeling method was used (TUNEL Andy Fluor™ 488 Apoptosis Detection Kit, Catalog Number: A050, Lot No: AB2150A2, ABP Biosciences). Following the manufacturer’s protocol, tissue sections were deparaffinized, rehydrated, and subjected to TUNEL staining. Sections were then counterstained with Hoechst 33342 to visualize all nuclei, coverslipped, and examined under a fluorescence microscope. Apoptotic cells were identified as TUNEL-positive cells, exhibiting bright green fluorescence within follicular structures of the ovarian tissue. For quantification, 10 random fields per section were selected at 20× magnification. Cells with green fluorescent nuclei in follicles were counted as apoptotic, and the apoptotic index (AI) was calculated as follows: AI (%) = (Number of TUNEL-positive cells × 100) / Total number of cells.

### Immunohistochemical Staining

Immunohistochemical staining for caspase-3 was performed on serial sections of ovarian tissue using the avidin–biotin–peroxidase (ABC) complex method. A dilution of 1:100 of the primary antibody, Caspase-3 Rabbit Polyclonal Antibody (Catalog No.: FNab01289, Lot No.: 20211215; FineTest), was used. For the negative control group, normal blocking serum was applied instead of the primary antibody. Following the staining procedure, immunoreactivity was evaluated based on the intensity of brown cytoplasmic staining. Dark brown staining was considered to be strong immunoreactivity (+++); light brown staining was considered to be weak immunoreactivity (+); and an intermediate staining intensity was considered to be moderate immunoreactivity (++). Expression levels were then assessed semi-quantitatively within groups using the H-score (H-SCORE = ∑Pi(I + 1)), where ‘I’ represents staining intensity (0 = none; 1 = weak; 2 = moderate; 3 = strong) and ‘Pi’ indicates the percentage of cells stained at each intensity level.

### Statistical Analysis

Primordial, primary, secondary, and tertiary follicle counts were performed on the ovarian sections of all subjects. Differences between groups were analyzed using the Kruskal-Wallis test, and differences between two groups were analyzed using the Mann-Whitney U test. Statistical analyses were performed by IBM SPSS Statistics for Windows, version 26 (IBM Corp., Armonk, N.Y., USA).

## Results

### Isolation and Characterization of OSSCs

**Fig. 2:**
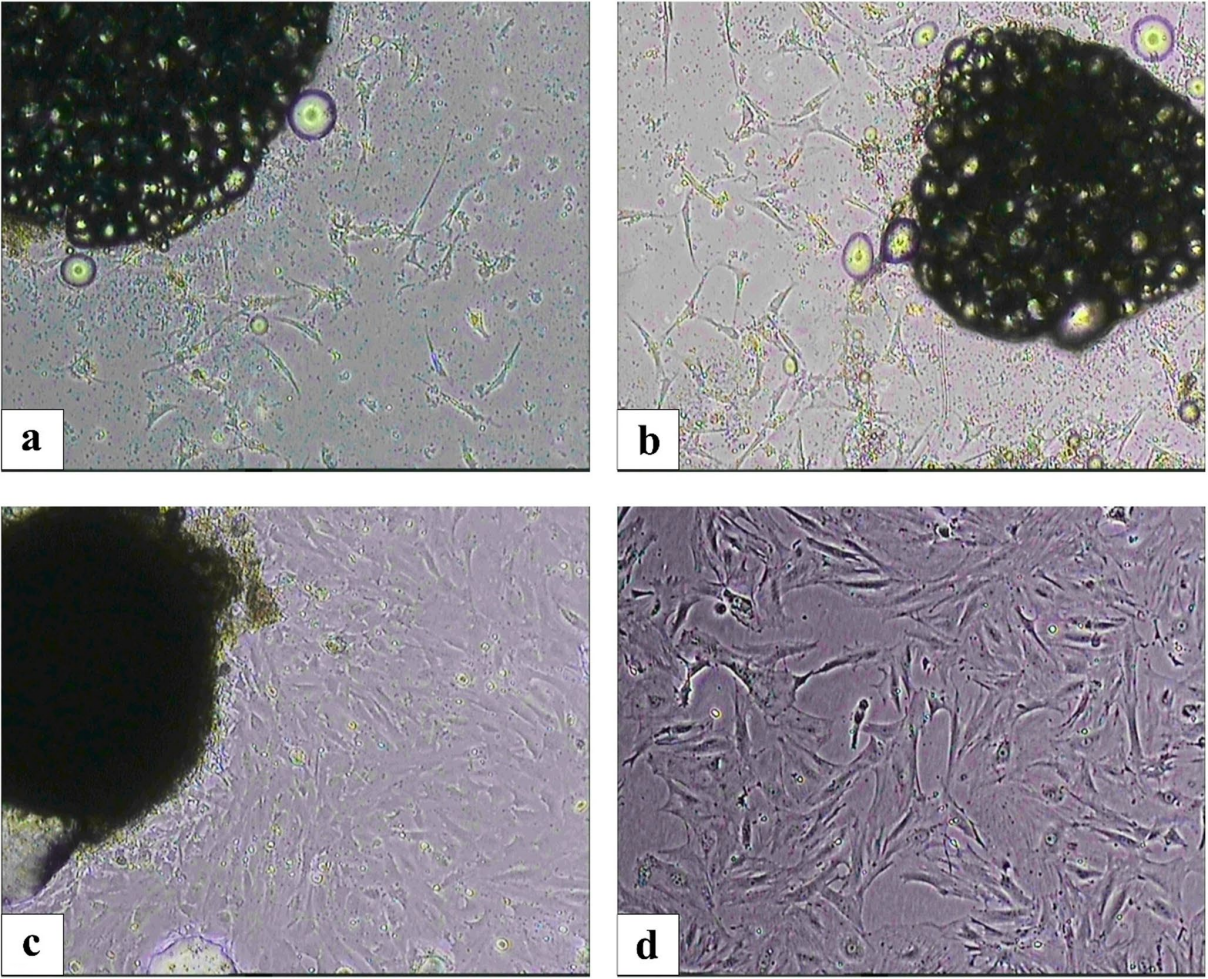
Morphological and phenotypic characterization of OSSCs. (**a**) On the 3rd day (**b**) On the 5th day (**c**) On the 9th day (**d**) After the 2nd passage

**Fig. 3:**
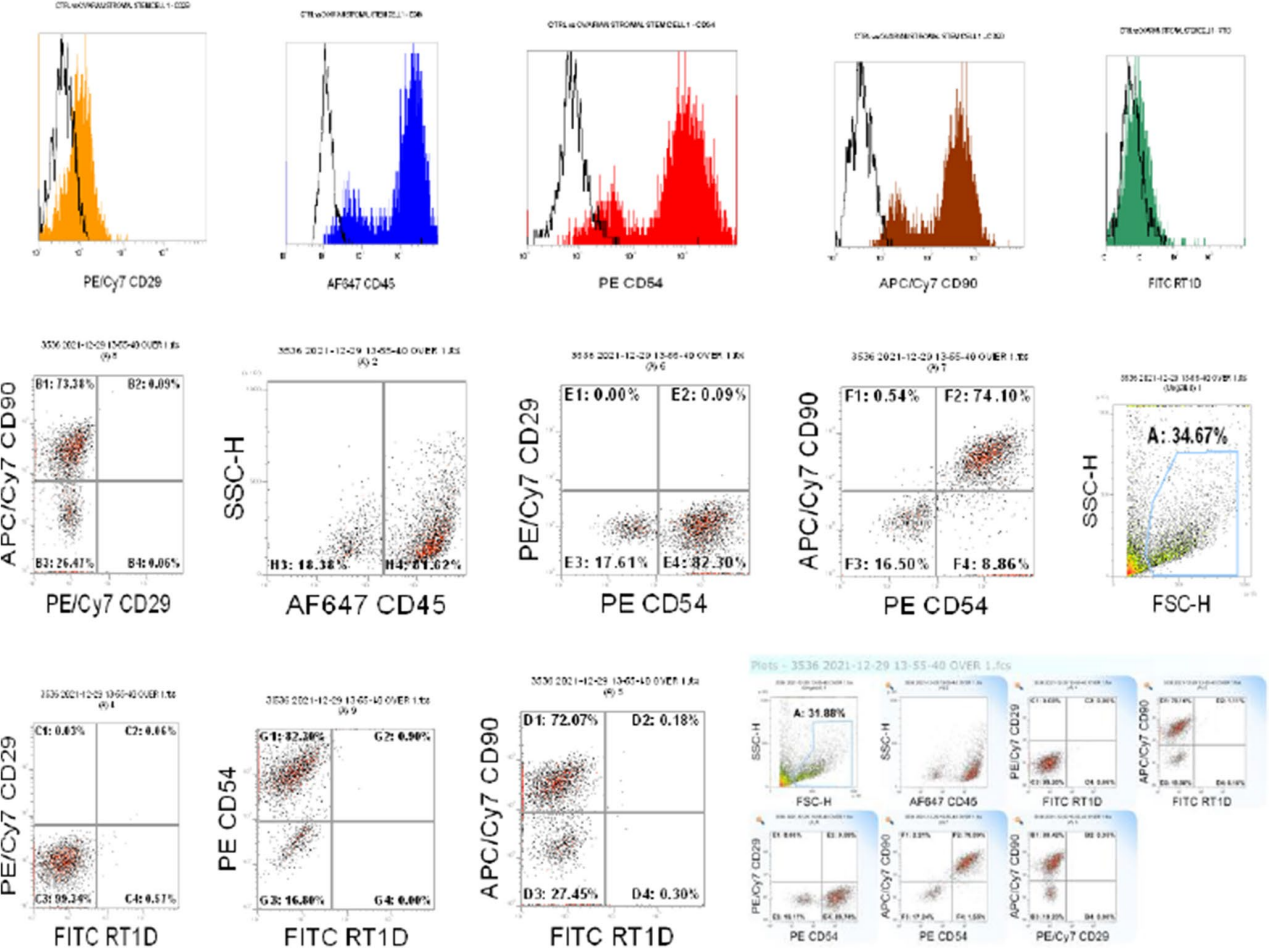
Characterization of undifferentiated OSSCs by flow cytometry showed that the cells expressed CD54 and CD90, exhibited lower levels of CD29 expression, and expressed CD45, a hematopoietic stem cell marker

**Fig. 4:**
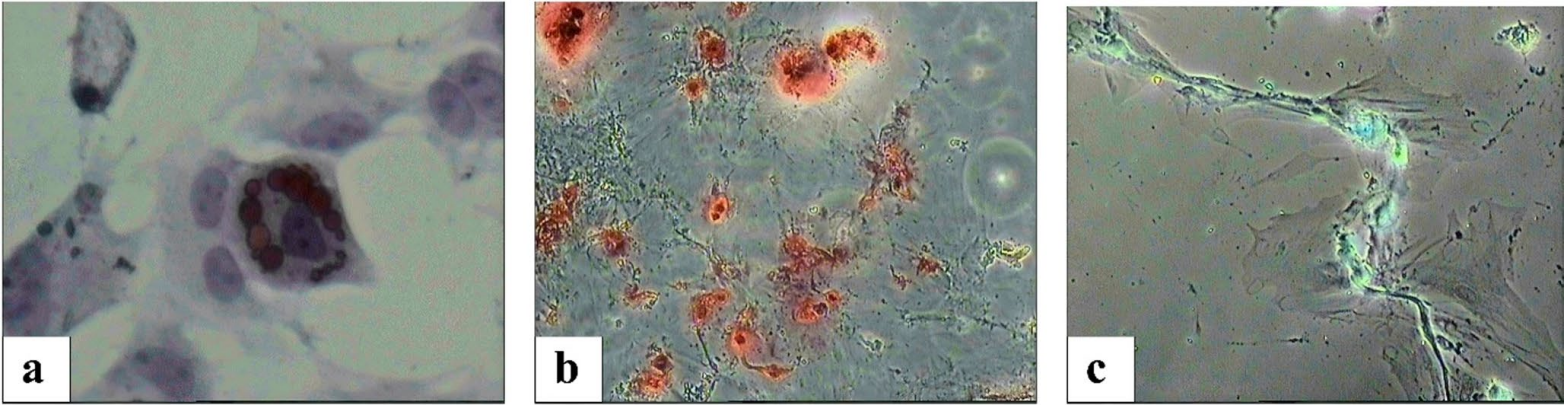
In vitro multilineage differentiation potential of ovarian stromal stem cells (OSSCs). (**a**) Adipogenic differentiation of OSSCs demonstrated by intracellular lipid droplet accumulation visualized with Oil Red O staining (×10). (**b**) Osteogenic differentiation confirmed by calcium deposition detected with Alizarin Red S staining (×4). (**c**) Chondrogenic differentiation evidenced by glycosaminoglycan-rich extracellular matrix stained with Alcian Blue (×10)

Ovarian stromal cells and ovarian surface epithelium were isolated from rat ovarian tissue using the explant culture technique without any enzymatic method. The stromal cells migrated from the tissues and adhered to the culture dishes on day 2, proliferating to reach confluency (70–80%) by day 7. When the morphology of the stromal cells in the culture medium was examined under a phase-contrast microscope, they appeared fusiform, spindle-shaped, and fibroblast-like (Fig. 2). Flow cytometry analysis showed the expression of CD54, CD90, and CD45 surface antigens, while CD29 was expressed at a lower level (Fig. 3). The multilineage differentiation capacity of the cultured cells was demonstrated through adipogenic, osteogenic, and chondrogenic induction. Following lineage-specific induction, adipocyte formation was verified by Oil Red O staining, osteoblast differentiation by Alizarin Red S staining, and chondrocyte formation by Alcian Blue staining. These staining results confirmed the ability of OSSCs to differentiate into adipogenic, osteogenic, and chondrogenic lineages under appropriate in vitro conditions (Fig. 4).

### Assessment of Body Weights

**Fig. 5:**
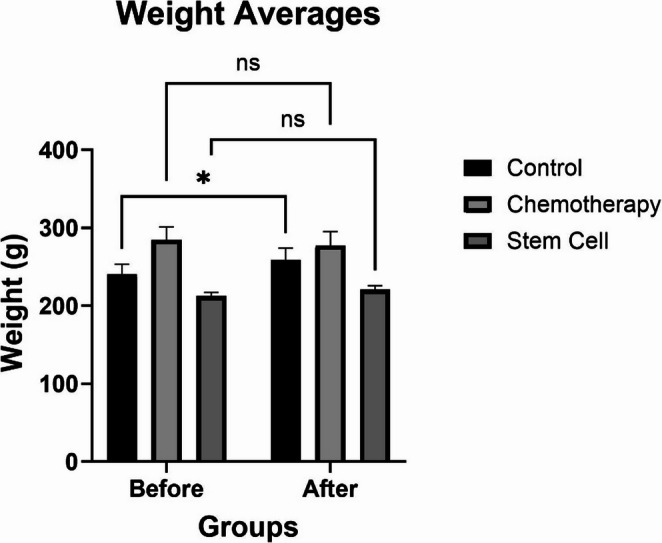
Comparison of body weight averages before and after treatment in the control, chemotherapy, and stem cell groups

Within-group comparisons of pre- and post-experiment body weights show increases in the control (*p* = 0.0795) and stem cell (*p* = 0.6721) groups; however, these changes were not statistically significant. In the chemotherapy group, although a decrease in body weight was observed after the experiment, this change was also not statistically significant (*p* = 0.7277) (Fig. 5 and Table 1).

**Table 1:** Body weight measurements in experimental groups

Group	Time Point	Mean (g)	Standard Deviation	Min-Max (g)	*p* value	
Control (*n* = 6)	Before	240.17	13.11	230–261	*p* > 0,005	
	After	258.17	15.87	242–283		
Chemotherapy (*n* = 6)	Before	284.50	16.79	262–300		
	After	277.17	18.15	252–296		
Stem Cell (*n* = 6)	Before	212.67	4.68	207–218		
	After	220.67	5.05	215–228		

### H&E Findings

**Fig. 6:**
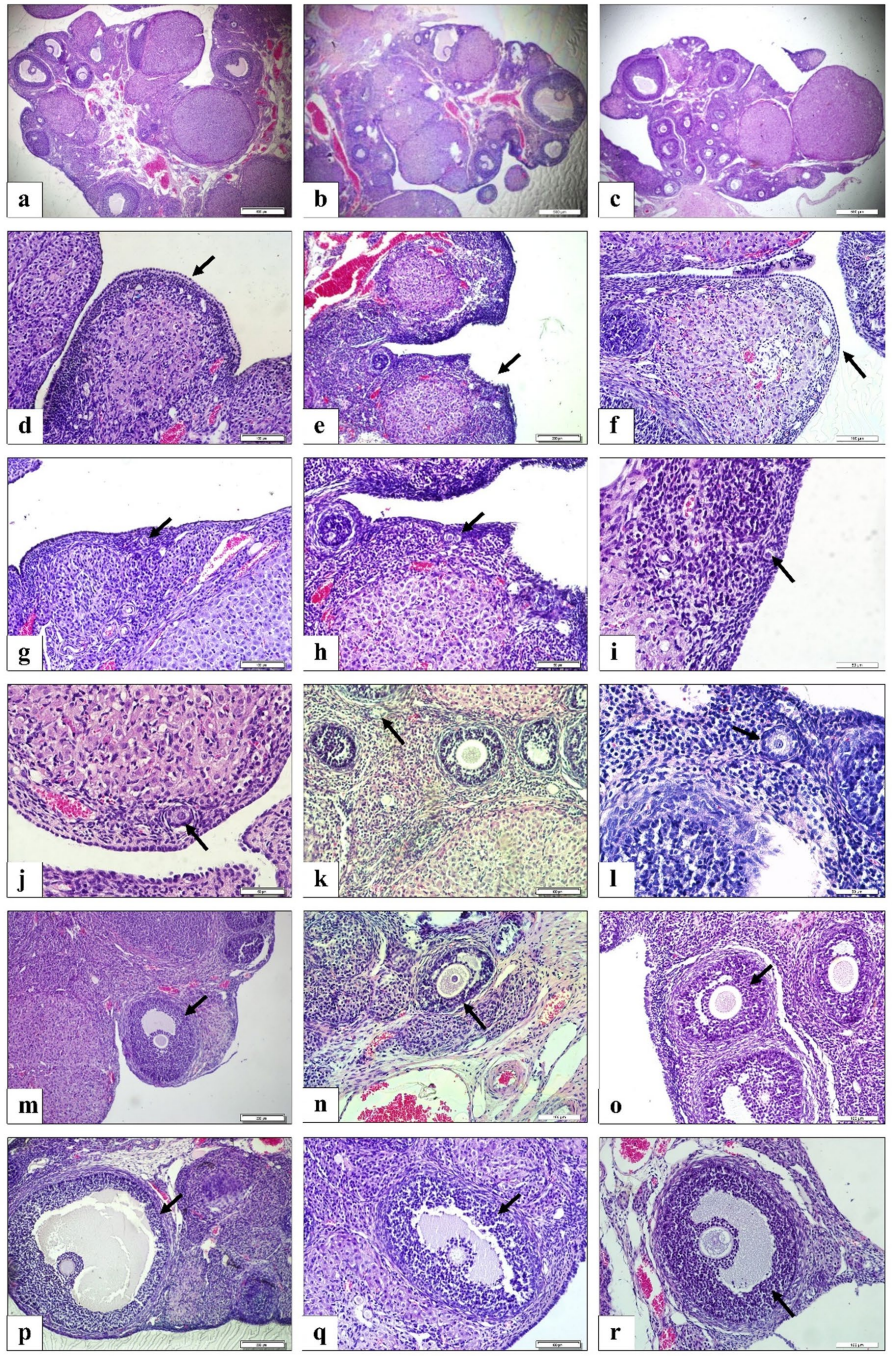
Histological images of ovarian tissue sections from the control, chemotherapy, and stem cell treatment groups under light microscopy (Hematoxylin and Eosin staining). **a**–**c**) General view of ovarian tissue in the control (**a**), chemotherapy (**b**), and stem cell treatment (**c**) groups. **d**–**f**) Surface epithelium integrity in the control (**d**), chemotherapy (**e**), and stem cell (**f**) groups (indicated by arrows). **g**–**i**) Primordial follicles observed in the control (**g**), chemotherapy (**h**), and stem cell (**i**) groups (indicated by arrows). **j**–**l**) Primary follicles in the control (**j**), chemotherapy (**k**), and stem cell (**l**) groups (indicated by arrows). **m**–**o**) Morphology of secondary follicles in the control (**m**), chemotherapy (**n**), and stem cell (**o**) groups (indicated by arrows). **p**–**r**) Graafian follicles in the control (**p**), chemotherapy (**q**), and stem cell (**r**) groups (indicated by arrows)

In the control group, the ovarian surface epithelium was observed to be covered by simple cuboidal epithelium in some areas and simple squamous epithelium in others. The tunica albuginea was located beneath the germinal epithelium. Follicles at different stages of development were seen beneath the tunica albuginea. Primordial follicles were identified in the cortical stroma. Secondary follicles, characterized by the formation of an antral cavity between the multiple layers of granulosa cells, were observed. In addition, tertiary follicles with a large single antrum were seen. Outside of the follicles, corpora lutea and atretic follicles were observed in different regions of the ovary. The medulla was noted to be rich in blood vessels, connective tissue cells, and fibers (Fig. 6).

In the chemotherapy group, it was observed that the integrity of the germinal epithelium was disrupted in some areas, while the tunica albuginea remained normal. Some follicles had lost their normal structure, with cytoplasmic loss in granulosa cells, and some granulosa cell nuclei appeared pyknotic. Atresia was particularly noted in secondary and Graafian follicles. Apoptotic granulosa cells were concentrated near the antral regions of secondary and tertiary follicles. Debris consisting of apoptotic granulosa cells was seen in the antrum of some follicles. Separation of stromal tissue was observed in certain areas. Atretic follicles displayed degenerated oocytes, zona pellucida, and vacuolization. The medulla showed noticeably dilated blood vessels (Fig. 6).

In the stem cell group, the overall morphology of the ovary appeared more similar to that of the control group. The integrity of the germinal epithelium, consisting of simple squamous epithelium in some areas and cuboidal epithelium in others, was preserved in the ovarian tissue. The tunica albuginea was present just beneath the epithelium. The number of healthy follicles in the cortex was higher compared to the chemotherapy group. Granulosa cells in the follicles appeared almost normal, and the natural structure of the cells was preserved. However, some separations in the stromal tissue were still observed. In the secondary and Graafian follicles, a more distinct theca layer was noted around the zona pellucida and granulosa cells (Fig. 6).

**Fig. 7:**
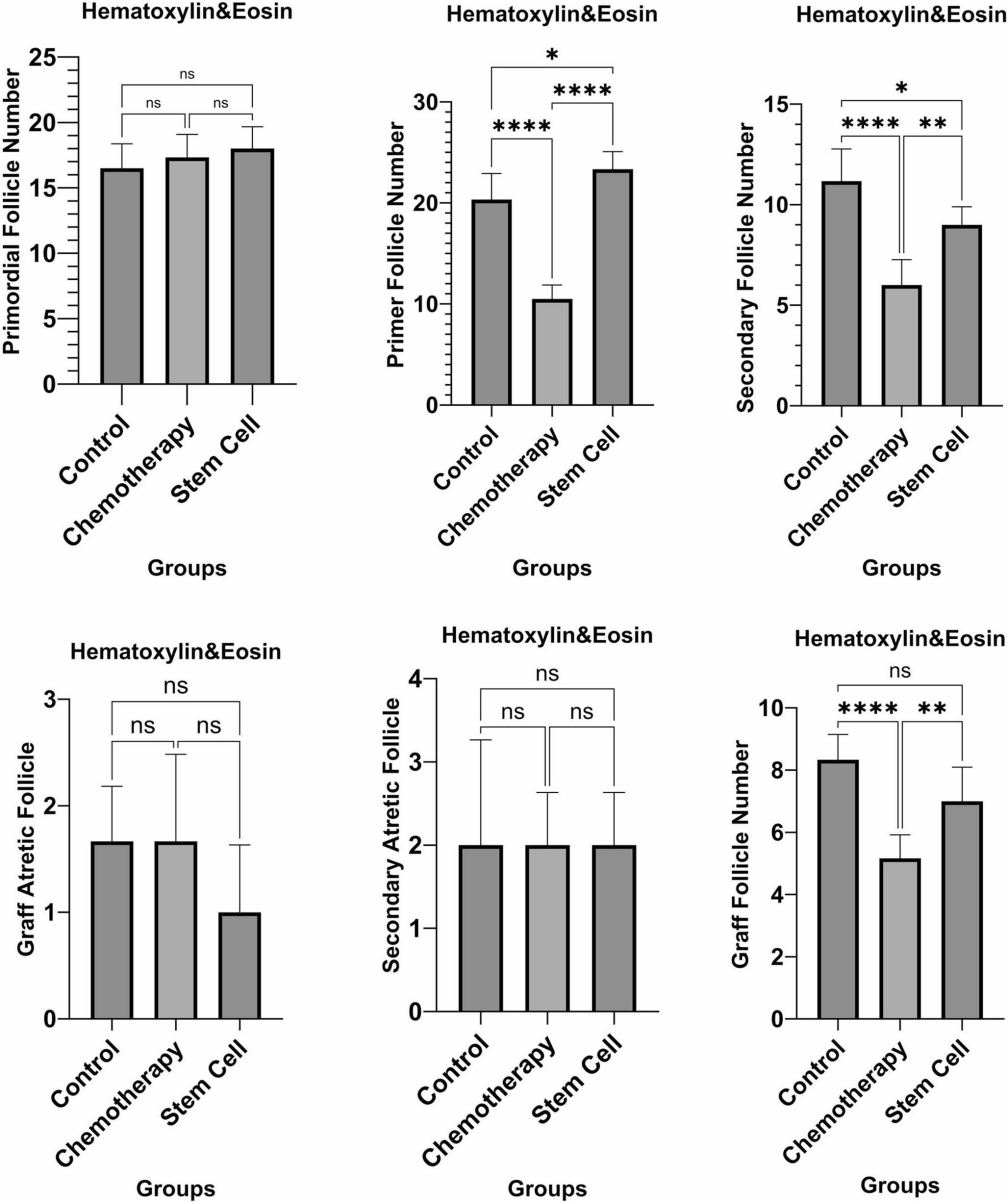
Distribution of the number of ovarian follicles according to groups

Upon statistical comparison of follicle counts between the groups, differences were detected in the numbers of primordial, secondary, Graafian, secondary atretic, and Graafian atretic follicles. No statistically significant difference was found in pairwise comparisons between groups for primordial follicles (*p* > 0.05). In primary follicles, the pairwise comparisons between the control group and the chemotherapy group (*p* = 0.001), the chemotherapy group and the stem cell group (*p* = 0.001), and the control group and the stem cell group (*p* = 0.0462) were statistically significant. In the comparison of secondary follicles between groups, a statistically significant difference was observed between the control group and the chemotherapy group (*p* = 0.001), the chemotherapy group and the stem cell group (*p* = 0.0029), and the control group and the stem cell group (*p* = 0.0271). In the pairwise comparisons of Graafian follicles, a statistically significant difference was detected between the control group and the chemotherapy group (*p* = 0.001), as well as between the stem cell group and the chemotherapy group (*p* = 0.0081). However, no significant difference was found between the control group and the stem cell group (*p* > 0.05). In the pairwise comparisons of secondary atretic follicles and Graafian atretic follicles, no statistically significant difference was detected between the groups (*p* > 0.005) (Fig. 7; Table 2).

**Table 2:** Follicular numbers in experimental groups

Follicle Type	Control (Mean ± SD)	Chemotherapy (Mean ± SD)	Stem Cell (Mean ± SD)	*p* (A-B)	*p* (B-C)	*p* (A-C)
Primordial follicle number	16.5 ± 1.870	17.3 ± 1.751	18.0 ± 1.673	> 0.05	> 0.05	> 0.05
Primary follicle number	20.3 ± 2.581	10.5 ± 1.378	23.3 ± 1.751	0.001	0.001	0.0462
Secondary follicle number	11.16 ± 1.602	6.0 ± 1.264	9.0 ± 0.894	0.001	0.0029	0.0271
Graaf atretic follicle	8.33 ± 0.816	5.16 ± 0.752	7.0 ± 1.09	> 0.05	> 0.05	> 0.05
Secondary atretic follicle	2.0 ± 1.264	2.0 ± 0.632	2.0 ± 0.632	> 0.05	> 0.05	> 0.05
Graaf follicle number	1.672 ± 0.516	1.672 ± 0.816	12.0 ± 0.632	0.001	0.0081	> 0.05

Note: A = Control group, B = Chemotherapy group, C = Stem cell group.

The *p* values for A-B, B-C and A-C comparisons are clearly shown in each cell.

Values with *p* < 0.05 are statistically significant, *p* > 0.05 are not significant.

### TUNEL Assay Findings

**Fig. 8:**
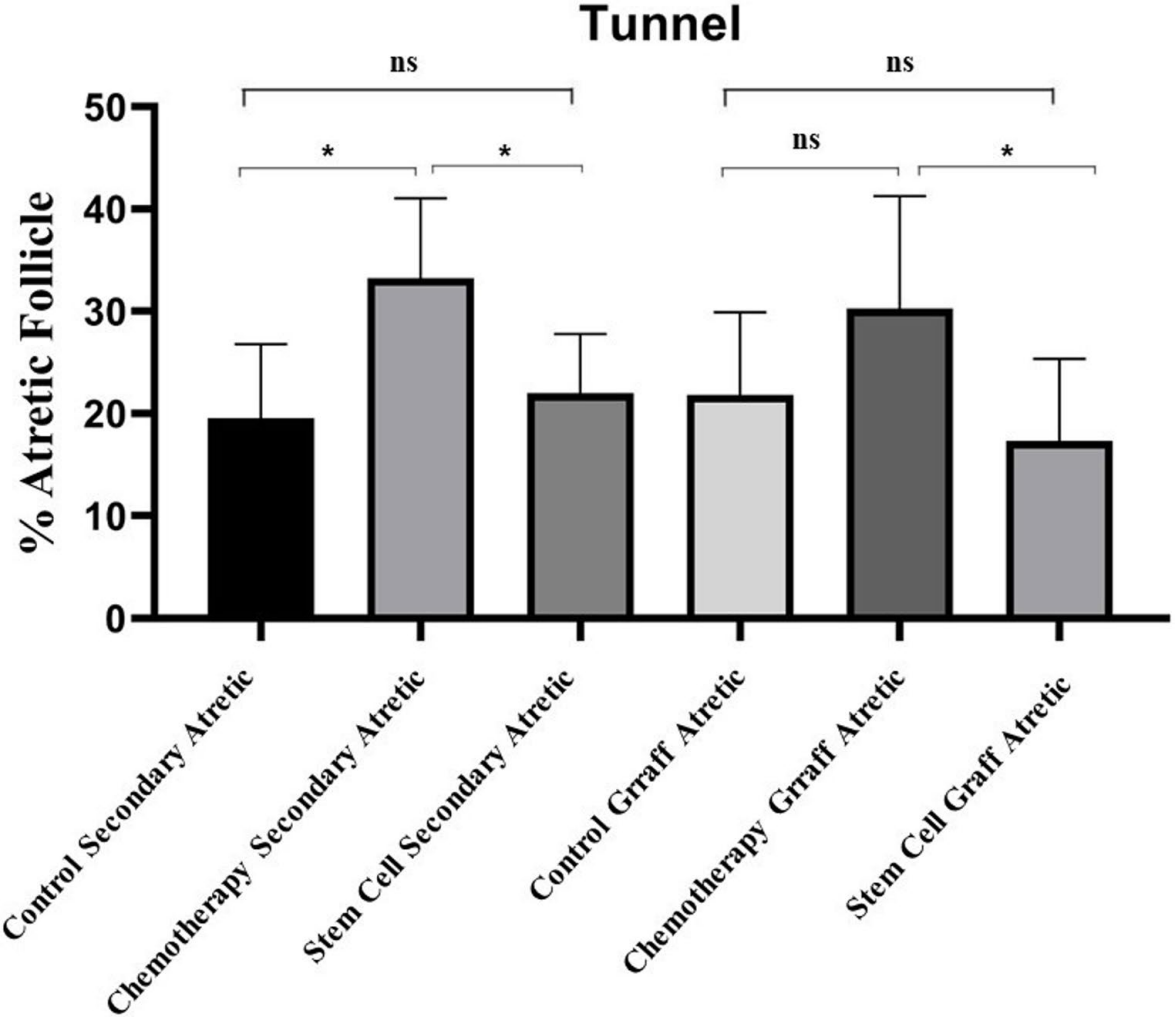
Comparison of apoptotic index (AI) in ovarian follicles using the TUNEL method

**Fig. 9:**
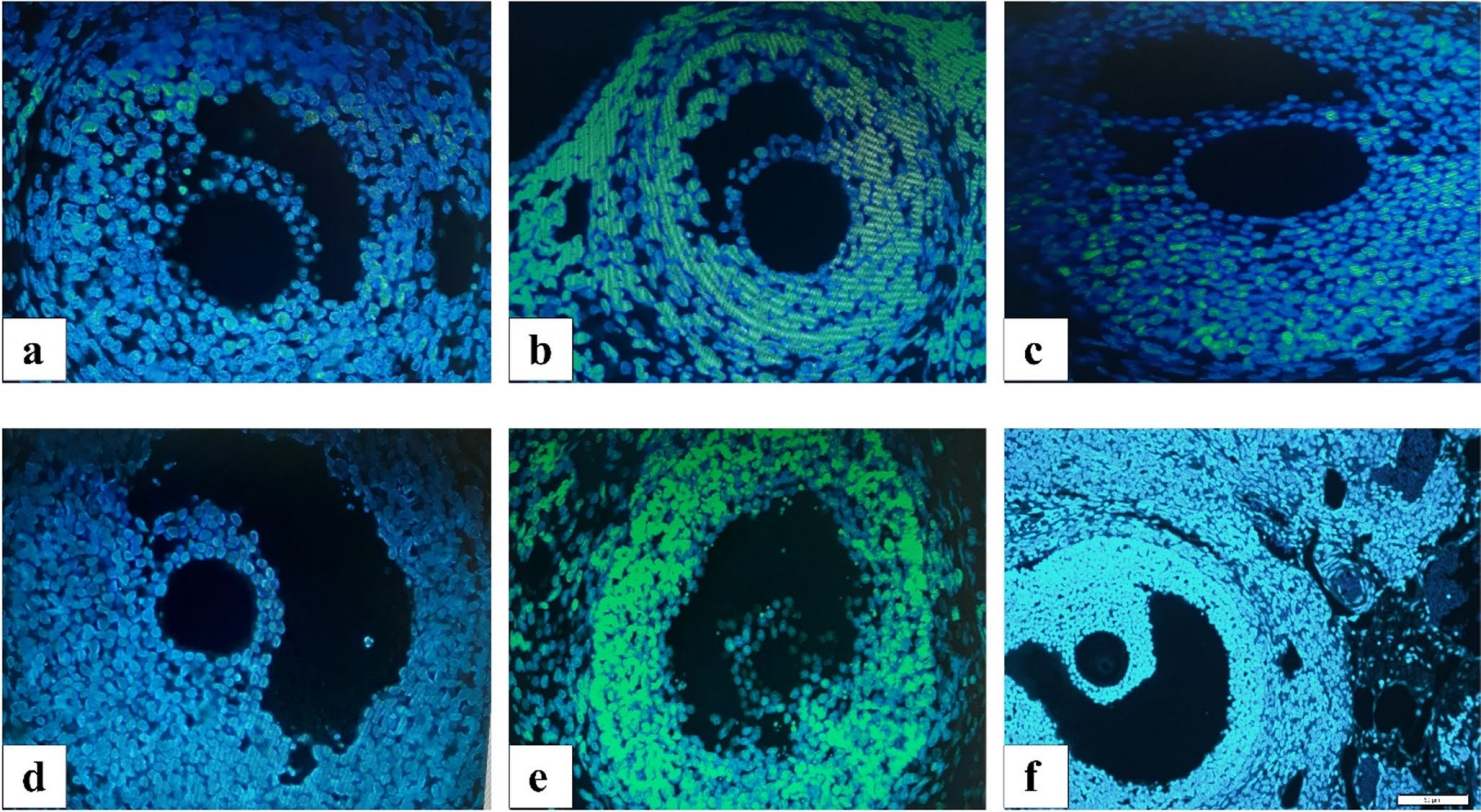
TUNEL assay images demonstrating apoptotic cell distribution within ovarian follicles across experimental groups. **a**–**c**) Secondary follicles from the control (**a**), chemotherapy (**b**), and stem cell-treated (**c**) groups. **d**–**f**) Graafian follicles from the control (**d**), chemotherapy (**e**), and stem cell-treated (**f**) groups

Statistical comparisons of the apoptotic index (AI) evaluated using the TUNEL method in ovarian tissues from all groups, performed with the Kruskal-Wallis test, revealed significant differences between the groups. In tissue sections from the ovaries, when secondary follicles were compared between the control group and the chemotherapy group, the AI was found to be statistically significantly higher in the chemotherapy group (*p* = 0.0173). No statistically significant difference in AI was observed in the pairwise comparison between the control group and the stem cell group (*p* > 0.05). In the pairwise comparison between the chemotherapy group and the stem cell group, the AI was found to be statistically significantly higher in the chemotherapy group (*p* = 0.0498) (Figs. 8 and 9).

In ovarian tissue sections obtained from Graafian follicles, no statistically significant difference was observed in AI between the control and chemotherapy groups (*p* > 0.05). No statistically significant difference in AI was observed in the pairwise comparison between the control group and the stem cell group (*p* > 0.05). In the pairwise comparison between the stem cell group and the chemotherapy group, the AI was found to be statistically significantly higher in the chemotherapy group (*p* = 0.0152) (Figs. 8 and 9).

In conclusion, apoptotic activation in the ovarian cortex and medullary stroma, as well as in the granulosa cells of developing follicles, was statistically significantly increased in the chemotherapy group compared to the other groups. In rats that received stromal stem cells derived from ovarian tissue after cyclophosphamide administration, apoptotic activation in the ovarian cortex and medulla decreased, with an increase in follicle numbers and a decrease in atretic follicle numbers (Figs. 8 and 9).

### Immunohistochemical Findings

**Fig. 10:**
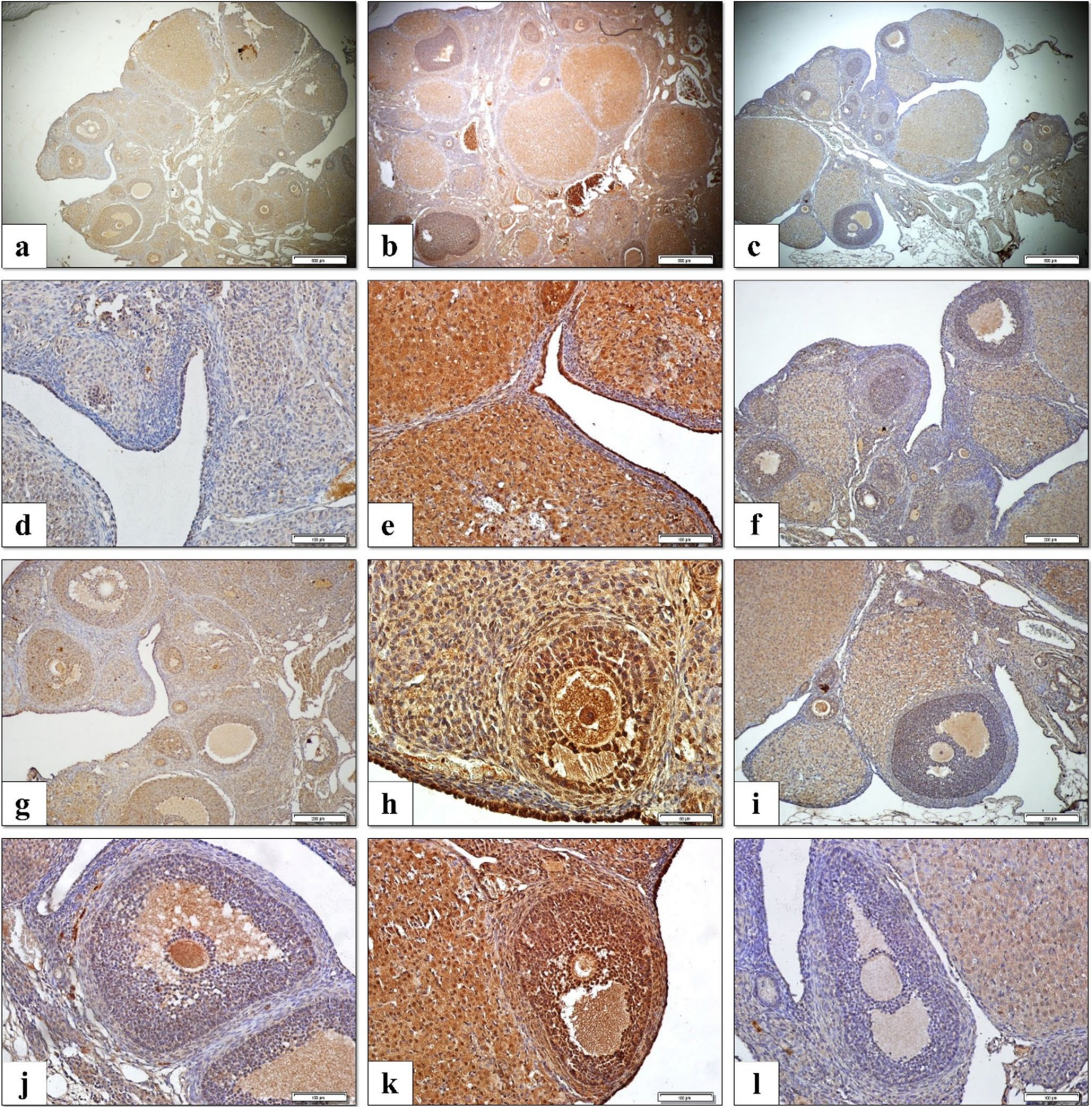
Immunohistochemical detection of Caspase-3 expression in FFPE ovarian tissue sections across experimental groups. **a**–**c**) Representative low-magnification images of ovarian tissue from the control (**a**), chemotherapy (**b**), and stem cell (**c**) groups. **d**–**f**) Caspase-3 expression in the surface epithelium of the control (**d**), chemotherapy (**e**), and stem cell (**f**) groups. **g**–**i**) Secondary follicles from the control (**g**), chemotherapy (**h**), and stem cell (**i**) groups showing varying levels of Caspase-3 immunoreactivity. **j**–**l**) Graafian follicles from the control (**j**), chemotherapy (**k**), and stem cell (**l**) groups demonstrating differential Caspase-3 staining

**Fig. 11:**
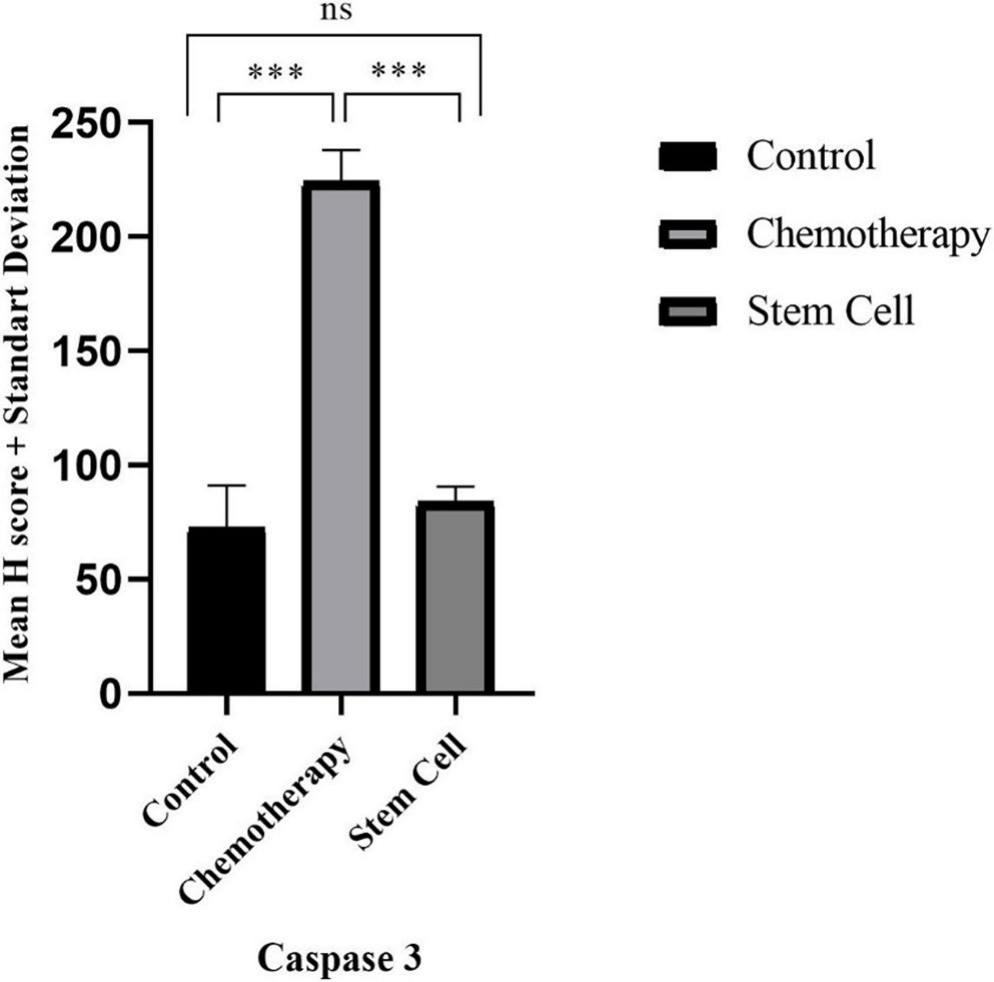
Graphical representation of caspase-3 H-score analysis across control, chemotherapy, and stem cell groups

In the control group, both granulosa and theca cells exhibited weak cytoplasmic immunoreactivity for caspase-3, and H-scores were correspondingly low. In contrast, in the chemotherapy group, strong caspase-3 expression was observed in both granulosa and theca cells, characterized by intense cytoplasmic staining, particularly within the follicular compartments. The H-score in this group was significantly higher compared to both the control and stem cell groups (*p* < 0.0001). In the stem cell group, moderate cytoplasmic caspase-3 expression was detected, primarily in scattered granulosa cells and, to a lesser extent, in theca cells. Compared to the chemotherapy group, the stem cell group exhibited a statistically significant reduction in H-score values (*p* < 0.0001) (Figs. 10 and 11).

## Discussion

The ovary consists of two primary components: parenchyma and stroma. While follicles constitute the parenchyma, the stroma serves as the supporting structure for the follicles. Ovarian follicles are predominantly located within the cortical stroma. The morphology of stromal cells differs between the cortex and medulla. Stromal cells, which resemble fibroblasts, are believed to originate from a mesenchymal cell population. In the cortex, these cells are arranged parallel to the surface and exhibit a rounded morphology, whereas in the medulla, they are randomly organized and possess an elongated structure. Notably, stromal cells do not form a single homogeneous population [12, 13].

Given the heterogeneous nature of stromal cells, a more detailed characterization is essential to distinguish their subpopulations and functional properties. In this study, flow cytometry analysis confirmed the identity of the isolated ovarian-derived stromal stem cells, providing further insight into their mesenchymal-like characteristics. Flow cytometry analysis demonstrated that the isolated ovarian-derived stromal stem cells expressed CD90, CD54, and CD45 while CD29 was expressed at a lower level. Our findings were consistent with those of Altinbasak et al. [14]. Phenotypic characterization further revealed that these cells share fundamental traits with mesenchymal stromal cells (MSCs). Given the broad variability in CD surface marker expression among MSC populations, their molecular identification remains complex. Additionally, different MSC subpopulations, even when derived from the same tissue, may exhibit distinct differentiation capacities. In this regard, our findings align with previous studies, which have also reported similar marker profiles in ovarian stromal stem cells [15]. However, inconsistencies persist across the literature regarding the definitive set of markers required for precise characterization, highlighting the need for standardized identification criteria.

The origin of theca cells—whether they arise from cortical or medullary stromal cells—has long been a subject of investigation. Studies have shown that cortical stromal cells actively differentiate into theca cells in the presence of granulosa cells. Growth differentiation factor-9 (GDF-9), which is derived from the oocyte, plays a crucial role in promoting the differentiation of theca cells [16, 17].

Follicular development in the ovary occurs through cell-cell interactions, as well as endocrine, autocrine, and paracrine signaling mechanisms. The pathways regulating the transition from primordial to primary follicles must function in a delicate balance, acting as both activators and suppressors to maintain the follicular pool [18, 19]. Disruptions in this balance can lead to premature activation of follicles, resulting in the rapid depletion of the follicular reserve [20].

One of the most significant side effects of chemotherapy is infertility and premature ovarian failure (POF). Preventing POF and preserving the ovarian follicle pool is crucial for improving the quality of life in female cancer patients undergoing chemotherapy. In young women, premature ovarian failure occurs in a substantial proportion of patients following treatment with cyclophosphamide [21].

Among the pathological alterations associated with primary ovarian insufficiency (POI) are ovarian tissue fibrosis and impaired follicular development. It has been demonstrated that transplantation of human umbilical cord-derived mesenchymal stem cells (hUMSCs) can restore ovarian function in rat models of POI. This improvement is associated with the inhibition of ovarian fibrosis through the regulation of ovarian stromal cell differentiation via the TGF-β1/Smad3 signaling pathway. Ovarian stromal cells were isolated from the ovarian cortex of 3- to 4-week-old immature rats, and these cells were shown to express vimentin. In vitro, human umbilical cord-derived mesenchymal stem cells have been shown to play a role in the differentiation of ovarian stromal cells into theca cells via the TGF-β1/Smad3 signaling pathway, thereby preventing fibrosis [22]. Similar to these findings, in a study conducted by Ünal et al., heterotopic ovarian transplantation was performed using periovarian adipose tissue without the use of any scaffold or bioengineering method. They reported that periovarian adipose tissue effectively prevented post-transplantation ischemia, preserved both follicular and stromal cell integrity, and prevented fibrosis in the ovarian parenchyma. These findings emphasize the intrinsic protective role of periovarian adipose tissue in maintaining ovarian follicle reserve under ischemic stress conditions [23]. In our previous studies, we found that ovarian stromal stem cells could not be effectively isolated from rats aged 12–14 months, corresponding to the advanced reproductive stage. Moreover, the few ovarian stromal cells that were isolated failed to proliferate and did not expand sufficiently to allow passaging. These observations led us to speculate that the cells present at this stage may have lost their capacity for asymmetric division and, consequently, their ability for self-renewal (unpublished data).

Numerous studies have experimentally induced ovarian failure in animal models and subsequently administered MSCs derived from various sources. Zheng et al. administered human umbilical cord-derived MSCs to rats with cyclophosphamide-induced ovarian failure and investigated the NGF/TrkA signaling pathway. Their findings indicated that MSCs contributed to ovarian microenvironment repair and folliculogenesis via this pathway [24]. Similarly, Beşikcioğlu et al. demonstrated that ovarian stromal stem cells exhibited superior efficacy compared to bone marrow-derived MSCs (BM-MSCs) in restoring ovarian function in a rat model of cyclophosphamide-induced ovarian failure [11].

Hormonal parameters are considered important biomarkers for evaluating ovarian function. In particular, anti-Müllerian hormone (AMH) is regarded as an indicator of follicular reserve, whereas follicle-stimulating hormone (FSH) and estradiol (E2) levels reflect the endocrine function of the ovary. In this context, in our previous study accepted for publication, we investigated the effects of ovarian stromal stem cells (OSSCs) in a rat model of ovarian aging using 12–14-month-old Sprague–Dawley rats with regular estrous cycles and low oocyte numbers. In our study, OSSC transplantation showed a tendency to increase AMH levels compared with the control group, while FSH levels remained comparable to those of the control group. In contrast, E2 levels showed a statistically significant increase. These findings suggest that OSSCs may support the endocrine function of ovarian tissue and have the potential to enhance follicular activity. Although hormonal parameters were not evaluated in the present study, these previous findings support the therapeutic potential of OSSCs in preserving ovarian function.

Ding et al. reported that human amniotic mesenchymal stem cells ameliorated the effects of natural ovarian aging in mice by secreting hepatocyte growth factor (HGF) and epidermal growth factor (EGF) [25]. Takehara et al. found that adipose-derived mesenchymal stem cells (AD-MSCs) facilitated ovarian regeneration in rats with cyclophosphamide-induced ovarian failure, suggesting their potential utility in regenerative medicine [26]. Yang et al. demonstrated that microvesicles secreted by human umbilical cord-derived MSCs promoted angiogenesis and ovarian recovery in mice [27].

Liu et al. administered human amniotic fluid-derived MSCs to mice with ovarian insufficiency and observed enhanced follicular development, granulosa cell proliferation, and secretion activity [28]. Mohamed et al. transplanted bone marrow-derived MSCs into mice with ovarian failure induced by cyclophosphamide and busulfan, noting restoration of folliculogenesis and ovarian hormone production [29]. Li et al. showed that human umbilical cord-derived MSCs improved ovarian function in perimenopausal rats via a paracrine mechanism, as evidenced by increased expression and protein levels of HGF, vascular endothelial growth factor (VEGF), and insulin-like growth factor (IGF-1) [30].

Song et al. administered human umbilical cord-derived MSCs to rats with cyclophosphamide-induced ovarian failure, reporting improved hormone secretion and folliculogenesis, as well as reduced apoptosis in ovarian cells [31]. Similarly, Huang et al. demonstrated that exosomes derived from adipose tissue-derived MSCs enhanced ovarian function via the SMAD signaling pathway in mice with cyclophosphamide-induced ovarian failure [32]. Liu T et al. found that human amniotic fluid-derived MSCs, when transplanted into mice with premature ovarian failure, maintained viability and proliferative capacity [33].

The use of chemotherapeutic agents activates the mTOR signaling pathway in the ovaries, thereby contributing to follicular depletion. The mTOR (mammalian target of rapamycin) pathway plays a pivotal role in cell growth, proliferation, metabolism, and angiogenesis. It consists of two distinct complexes: mTORC1 and mTORC2. Studies indicate that the mTOR pathway regulates granulosa cell proliferation and differentiation within ovarian follicles. Excessive activation of this pathway has been implicated in polycystic ovary syndrome (PCOS) and ovarian cancer [34–37]. Rapamycin, an inhibitor of the PI3K/Akt/mTOR signaling pathway, has been shown to counteract cyclophosphamide-induced follicular depletion by preventing primordial follicle activation, thereby preserving the follicular pool [38–41]. In the present study, apoptosis was evaluated using both cleaved caspase-3 immunostaining and the TUNEL assay to detect different stages of apoptotic cell death. Caspase-3 is synthesized in the cytoplasm as an inactive proenzyme and becomes activated through proteolytic cleavage by initiator caspases such as caspase-8 and caspase-9 in response to intracellular apoptotic signals. The cleaved (active) form of caspase-3 is localized mainly in the cytoplasm and represents an indicator of early apoptotic activation [42]. In contrast, the TUNEL assay detects DNA fragmentation, a hallmark of late-stage apoptosis, with staining typically observed in the nucleus [43]. Therefore, the combined use of these two techniques allowed us to identify both early and late apoptotic cells, providing a more comprehensive assessment of apoptosis in ovarian tissue.

Although the findings of this study support the therapeutic potential of ovarian stromal stem cells in mitigating chemotherapy-induced ovarian damage, several limitations must be acknowledged. Since it focused on the acute effects of chemotherapy on ovarian tissue, only apoptotic markers were analysed. In future studies, the long-term effects of ovarian stromal stem cells should be evaluated, and hormone levels such as AMH, FSH, and E2 should be measured to better understand their therapeutic potential. Furthermore, oxidative stress plays a pivotal role in chemotherapy-induced ovarian damage; however, in this study, oxidative stress parameters such as reactive oxygen species (ROS) levels, lipid peroxidation markers (e.g., MDA), and antioxidant defense indicators (e.g., GSH, SOD) were not evaluated. Future studies should incorporate these parameters to gain a more comprehensive understanding of the protective mechanisms of ovarian stromal stem cells.

## Conclusion

In our study, it was observed that (allogeneic) ovarian stromal stem cell treatment in rats that received chemotherapy reduced the number of atretic follicles in the ovaries and increased the number of normally developing follicles. This increase was similar to the results of the control group and rats that received ovarian tissue-derived stromal stem cells. It was observed that ovarian tissue-derived stromal stem cell treatment reduced the tissue damage and follicular loss caused by cyclophosphamide in the ovary. Ovarian tissue-derived stromal stem cell treatment applied after chemotherapy was effective in protecting both developing follicles and the primordial follicle pool. Stromal stem cell application preserves the normal structure of both primordial follicles and developing follicles, allowing them to reach ovulation. Our study is a study showing that ovarian stromal stem cells have therapeutic effects in ovarian toxicity following cyclophosphamide, and may be an alternative treatment option to approaches aimed at preserving fertility.

Cryopreservation and subsequent transplantation of ovarian tissue currently represent an important option for fertility preservation in young female cancer patients. Among available methods, slow freezing of ovarian tissue is the most commonly used technique. Although this approach preserves follicles, it often results in damage to the stromal cells. Therefore, cryopreservation strategies developed for ovarian tissue should aim to ensure the preservation of all tissue components. In addition, more detailed knowledge of the characteristics of ovarian stromal cells may lead to the development of cryopreservation techniques for preserving fertility during the freezing and thawing of ovarian tissue.

## Data Availability

The data that support the findings of this study are available from the corresponding author upon reasonable request.
